# Is enough attention given to climate change in health service planning? An Australian perspective

**DOI:** 10.3402/gha.v7.23903

**Published:** 2014-06-18

**Authors:** Anthony J. Burton, Hilary J. Bambrick, Sharon Friel

**Affiliations:** 1 School of Medicine, University of Western Sydney, Campbelltown, Australia; 2 National Centre for Epidemiology and Population Health, Australian National University, Canberra, Australia

**Keywords:** climate change, health service planning

## Abstract

**Background:**

Within an Australian context, the medium to long-term health impacts of climate change are likely to be wide, varied and amplify many existing disorders and health inequities. How the health system responds to these challenges will be best considered in the context of existing health facilities and services. This paper provides a snapshot of the understanding that Australian health planners have of the potential health impacts of climate change.

**Methods:**

The first author interviewed (n=16) health service planners from five Australian states and territories using an interpretivist paradigm. All interviews were digitally recorded, key components transcribed and thematically analysed.

**Results:**

Results indicate that the majority of participants were aware of climate change but not of its potential health impacts. Despite this, most planners were of the opinion that they would need to plan for the health impacts of climate change on the community.

**Conclusion:**

With the best available evidence pointing towards there being significant health impacts as a result of climate change, now is the time to undertake proactive service planning that address market failures within the health system. If considered planning is not undertaken then Australian health system can only deal with climate change in an expensive ad hoc, crisis management manner. Without meeting the challenges of climate change to the health system head on, Australia will remain unprepared for the health impacts of climate change with negative consequences for the health of the Australian population.

Within an Australian context, the medium- to long-term health impacts of climate change are likely to be wide and varied. They will range from increased incidence of mental health issues (1), heat stress (2–4), and severe weather events (5) to expanded ranges of vector-borne disease and zoonosis (6, 7), a reduction in food and water quality (8), and an increase in both food and water-borne disease (8–10). Furthermore, climate change will exacerbate air pollution and increase aeroallergens which will likely lead to an increase in asthma-related disorders (11, 12). While not specifically set in an Australian context, Nilsson et al. suggested that climate change will constitute a public health threat ‘at least as wide-ranging as the effects of tobacco on health’ (13). Rather than heralding new diseases, climate change is likely to amplify many existing disorders and health inequities (14, 15) and therefore health system responses will be best considered mostly in the context of existing health facilities and services (15, 16).

The types and intensity of impacts will vary according to region and unevenly affect population sub-groups, reflecting the influence of environment, socio-economic circumstances, infrastructural and institutional resources, underlying physical vulnerabilities such as age, ethnicity, socioeconomic status and chronic disease, and local preventive (adaptive) strategies (17). But it will not just be the vulnerable who will be impacted by climate change; events such as Hurricane Katrina in the USA, the European heat waves of 2003, the Queensland floods of 2011 and 2013, and the record-breaking heat waves in south eastern Australia of 2013 and 2014 (18, 19) show that all societies are vulnerable to the new extremes in weather (19–22).

The health impacts of climate change will not occur all at once or in a predictable order, rather some impacts will become evident well before others. Some impacts will occur via direct and easily measurable pathways (17), while others will occur via indirect pathways entailing disturbances in natural ecological systems (6), failures of the urban planning system (23–26), or through changes in economic circumstances such as disruption to livelihoods and communities (27).

The likely medium- to long-term impacts of climate change on Australian populations will lead to an increased strain on aged care services, community-based services, primary health services, and ultimately an increase in demand for tertiary health-based services.

The structure of the current Australian health system is that it is largely funded at both state level and national level according to the Australian Health Care Agreements which are aimed at preventing illness and meeting the needs of the population and ensuring equity (28). Health service planning, however, occurs at state level with administration by the state governments and a small amount of planning at municipal level. Planning is the act of identifying the future health needs of a population and plans for new and aligns existing services that reflect this need while aiming to make the most effective use of available resources (29, 30).

Australian government policy on climate change has been categorised as inconsistent (31). However, current Australian government policy identifies that departments should embed climate change adaptation within their existing intuitional frameworks (32). They further identify that it is important to improve awareness and understanding and to increase the capacity of decision makers to plan for the impacts of climate change (32, 33). Therefore, to ensure that the health system is proactive with regards to climate change, those tasked with planning the future of the health system need to be aware and informed on the potential health issues that will arise as a result of climate change (34).

Within Australia, there are examples of inter-sectoral actions that target medium- to long-term climate impacts (35, 36); however, these examples are sporadic and have not been universally adopted within an Australian context. This response is perhaps reflective of Australian attitudes to climate change, which indicates that there is a belief that it is happening but that most people do not ‘consider it to be a threat to them personally’ (37). Thus, within the politicised nature of the health system, planning for climate change may not be seen as a high priority.

The authors present a case study exploring attitudes towards climate change among Australian health service planners, in particular their awareness of the potential health impacts of climate change and their perceived need to incorporate climate change into their health service plans.

## Methods

This research was undertaken using a qualitative research method employing a series of independent interviews under an interpretivist paradigm where the participants are seen as the experts in their field (38).

Prior to the interview, each participant was provided with a background paper prepared by the first author summarising current literature on health and climate change. Participants were encouraged to read the paper prior to interview but this was not compulsory. The background paper included the questions that formed the basis for the study and was provided so that all participants had a baseline knowledge of the subject and were able to prepare for the interview. Participants were asked to reflect on the background paper and respond to the question: ‘What do you see as the medium to long-term health impacts of climate change that your organisation will have to respond to?’ ‘Organisation’ here refers to the state or territory health services or private sector services that the participants were employed by.

The background paper included a list of likely health impacts identified by the Australia's Environmental Health Committee (9). These were:

1. Mental health
2. Heat stress
3. Severe weather events (other than heat events), for example, bushfire, storm, sea surge
4. Health services demand
5. Needs of vulnerable populations
6. Vector-borne disease and/or zoonoses
7. Aged care services demand
8. Food quality and safety
9. Water quality and/or safety
10. Air pollution and aeroallergens
11. Food availability

Interviews were conducted by the first author who was aware of and utilised the Whyte directiveness scale in an effort to reduce introduced bias during interviews (39).

Participants were from New South Wales, the Australian Capital Territory, Victoria, Tasmania, and Western Australia and were all either senior civil servants or senior representatives from private planning companies. Participants’ job titles included Senior Health Service Planner, Senior Planning Manager, and Executive Director of service planning units amongst others. Their specific job titles have not been included in this paper to ensure that their contributions remain anonymous. While relatively small in number (*n*=16), the interview sample represents a cross-section of senior Australian health service planners who are responsible for approval of plans which require the expenditure of millions of capital and recurrent dollars (40–42).

Participants were identified through snowball sampling (38) with a focus on health planners at a state and territory level who were generally planning for health service at a local/regional level. This reflects the structure of the Australian health system which is funded at both a state and national level but where services are administered by the state governments.

Forty-eight Australian health service planners were invited to participate and 16 accepted. Thirteen interviews were conducted by telephone and three were conducted face-to-face. The interviews were semi-structured using a conversational style with open-ended questions that allowed further exploration of particular issues that arose. Interview summaries and transcripts were de-identified so that participants could be candid with their answers and that privacy was maintained.

Interviews with Australian health service planners were conducted between September 2011 and February 2012.

All interviews were digitally recorded and key components of the answers were transcribed by the first author. Other components were recorded and summarised but not transcribed verbatim. A matrix of key impacts was developed identifying keywords and thematically analysed by the first author.

This study was approved by the University of Western Sydney Human Research Ethics Committee (approval number H9266).

## Results

Sixteen senior health planners were interviewed from five Australian states and territories.

From the information given by participants in interviews, attitudes to the role of health planners in responding to the likely impacts of climate change were categorised into three broad groups:

1. Those who were either sceptical or did not believe that climate change would have any impact on their work (*n*=2):

… from what I've seen at this stage nobody's identifying climate change as being a reason for any sort of increase in activity.

2. Those who, until being interviewed, were unaware or had not thought about the health impacts of climate change in the context of their work (*n*=12):

I suppose that it could be an issue but had never thought of it as such prior to reading the background paper, reading the questions and participating in this research.

3. Those who were well-informed and proactive in planning for the health impacts of climate change (*n*=2):

… people can't help but notice that something is happening and something has to be done. That's a big step forward.

In addition to those who actively participated in the research, a fourth group (made up of planners who were invited but chose not participate) could also be identified through responses obtained as part of the recruitment process:

4. Those who identified that climate change was outside of the scope and relevance of their current planning.

my area within (the de-identified health department) is not the appropriate area for [questions about climate change and health]

Table 1 presents a summary of participants’ responses to questions concerning the health impacts of climate change. The stacked bars show the total number of participants who either agreed that this is a likely impact that requires planning, disagreed that this is a likely impact that requires planning or did not identify the potential health impact during the interview.

**Table 1:** Participants’ perceptions about whether climate change impacts need to incorporated into their health service plans

Comment (themed, not transcribed)	Number of participants
Mental health	
Climate change will increase the numbers and complexity of mental health client within the community and will require a planned response from health services.	12
Climate change may impact upon the mental health of the community but will not require a planned response from health services.	4
Heat stress	
Australia is likely to experience more hot days and extended heat waves. This experience will require a specific planned response from health services.	11
Australia has always had hot days and heat waves and health services do not require a specific planned response to deal with the potential for additional heat waves.	4
Additional stress seen in health services is due to bed block and reduced summer services rather than heat stress. Climate change is not likely to affect that.	1
Severe weather events	
Climate change will lead to an increase in the number, frequency and severity of severe weather events. Health services will need to specifically plan for these and the resultant increase in health service demand.	11
Past severe weather events have not required a change to health planning and future events are therefore not likely to require this.	3
Health service demand	
As a result of climate change there will be an increase in health service demand that will require a planned response.	9
Climate change will lead to a shift from health care provided primarily as primary and community based care towards tertiary (hospital) level care, thus increasing both the cost of health care and the need to plan for and provide additional or modified services.	1
Health service demand will continue to increase but climate change will not be a key factor or driver in that change. Other factors including the ageing population will be principal in driving change.	2
Vector-borne disease and zoonoses	
Climate change will see an increase in the number and range of potential vectors and a commensurate increase in vector-borne disease and zoonoses as a result. This will therefore require a planned response from health services.	8
Vector-borne disease and zoonoses is a small problem that can be treated on a case-by-case basis. Even if climate change were to increase the numbers of vectors and their range it would not require a planned response.	6
Aged care	
Australia's ageing population will be significantly impacted by climate change therefore health services will need to plan specifically for the health impacts of climate change for this age group.	7
Aged care and the ageing population have nothing to do with climate change.	4
Food-borne illness	
Climate change is likely to see an increase in food-borne illness and health services will need to plan for this.	6
Climate change is likely to see an increase in food-borne illness and the health department will need to respond to this but the planning of these services is outside of the scope of current practice.	2
There is no need to plan health services for any potential increase in food-borne illnesses.	4
Water-borne disease	
Climate change is likely to see an increase in water-borne illness and health services will need to plan for this.	6
Climate change is likely to see an increase in water-borne illness and the health department will need to respond to this but the planning of these services is outside of the scope of current practice.	2
There is no need to plan health services for any potential increase in water-borne disease.	4
Air pollution and aeroallergens	
Presentations to health services as a result of an increase in air pollution and aeroallergens will require a planned response from health services.	6
Air pollution and aeroallergens exist now and it is unlikely that, as a result of climate change health services will need specific planning.	5
Food availability	
Food availability will be impacted upon by climate change and this will ultimately have a flow on effect to the health system. This impact will need to be planned for.	5
Food availability has little to do with the health system and therefore will not require any specific planning.	5
Other issues	
Health services will need to plan for an increase rate of skin cancer as a result of climate change.	1
An increase in the numbers of climate refugees to Australia, with this vulnerable population being particularly susceptible to infectious disease and mental health disorders as a result of their displacement.	1
Climate change is outside of the scope of service planning.	1*

* Not recorded as a participant as they chose not to participate as an interviewee for this project.

## Discussion

These results demonstrate that despite climate change being described as the biggest global health treat of the 21st century (43), there appears to be a poor understanding of the potential impacts of climate change on health systems. The majority of participants (*n*=12) was aware of climate change *per se* but did not identify climate change as a factor in past trends and further climate change was not included as a factor in future modelling of health service demand. Even when participants agreed to the potential for climate change to affect a named set of health outcomes, these outcomes were not widely considered relevant to health service planning despite the evidence that they will have an impact on the health of the population in coming years and decades (10, 25, 27, 44–55), and despite the recognition of the problem by governments in Australia for over 25 years (32, 46, 56).

Highly visible and reported impacts of climate change, in particular heat stress and severe weather events, were identified by the majority of participants; there was little understanding of the potentially more subtle impacts of climate change such as aeroallergens and the impact of climate change on older Australians. Of particular note was that 12 participants identified the impact of climate change on mental health. This is perhaps reflective of the growing recognition and training among health care professionals on mental health issues and the prevalence of mental health problems in the community (57) and the impact that additional strains, like climate change may have.

Health service planners have yet to incorporate the impacts of climate change into their computer modelling and therefore their planning. This, in part, is as a result of the perceived lack of data about the health impacts of climate change.

(The ageing population is) there in front of us, we can see it, we can feel it, we have got the data for it … but what we haven't been able to … or nobody has told us, is the impact of climate change on health service provision.

Due to the lack of data, participants were uncertain about how much of an impact climate change would have on the services for which they were planning; while they believed they should plan for things they considered certain, such as the ageing population, they considered that there was too much uncertainty about climate change impacts and it therefore could not be incorporated into service planning.

Although lack of precision in estimating the exact impacts and their magnitude remains a limitation of impacts modelling, the likelihood of some level of impact is well accepted by researchers and could be factored into service modelling. Other health sectors have developed climate change plans (58), but the majority of health service planners were not aware of the likely climate change-associated impacts on population health, and were therefore unable to incorporate the evidence into their service plans.

This lack of awareness persists despite the Australian government policy that identifies ‘(t)he process of embedding climate change in new policy reform will involve explicitly identifying climate change risks and ensuring appropriate account of their implications is taken in policy development and program delivery’ (32). It seems that information is either not reaching those tasked with health service planning or is not being acknowledged by planners, or is recognised but not yet able to be used by the planners as broader decisions and policy are made elsewhere.

Clearly there are significant risks to health service effectiveness if a proactive planning strategy is not put in place for the health impacts of climate change. As Lawrence puts it ‘(t)he direct and indirect effects of climate change on public health are more complex and pervasive than any other issue confronted by public health professionals (59)’. A failure to plan for climate change risks increasing future climate vulnerability (60).

With the best available evidence pointing towards there being significant health impacts as a result of climate change, now is the time to undertake proactive service planning that addresses market failures within the health system (29) such as the health impacts of climate change (61). When combined with supportive government policy and following the lead in tackling climate change for other sectors, like urban, transport and energy planning, a planned approach by the health system is clearly a ‘no regrets’ path that will ultimately reduce the impact of climate change on the health system (55).

Health service planning is reliant on empirical testing of hypotheses and requires time for monitoring potential impacts. This traditional approach to health service planning does not allow for the early implementation of adaptive strategies, which may be necessary to provide the best response to climate change (10, 62).

Planning for the health impacts of climate change is clearly a complex problem and it is possible that it is seen as too complex and is therefore too difficult for planners to know where to begin. Such a lack of ordered and advanced planning means that the impacts of climate change may instead risk being confronted in an ad hoc, crisis management manner. Without meeting the challenge of climate change to the health system head on, Australia will remain unprepared for the health impacts of climate change.

One approach to initiate necessary changes in planning practices towards one that considers the changing climate might be to concentrate on services already associated with the highly visible, direct, and readily observable impacts such as heat, flooding, bushfire, and storms. Extreme heat has the potential to have a massive impact on the community (1, 4, 49, 63–66). Several Australian states are in the process of developing and testing heat wave action plans. Health services planning would benefit from consideration of these and inclusion of specific service responses into their health plans, such as strategies for assisting chronic-disease patients during heat waves who may not be directly under the care of a service provider, that is, they are living at home (67, 68).

The impact of climate change on the mental health of the community was the only area that was identified by all participants and as such could be an appropriate issue with which to commence health service planning for climate change.

Raising awareness about findings from health impacts modelling research may increase a willingness to consider climate change in health planning, as lack of access to information was noted by participants as a reason for not incorporating these likely impacts into their plans. Providing data in a workable format for health planners so that it could readily be incorporated into health services modelling may be required.

Eagar et al. identified that planning is an on-going process of learning and adapting to change and its protagonists are often as much activists as they are problem solvers (29). Thus, unless the planner has a specific interest in and/or knowledge of the subject, in this case climate change, the planner is unlikely to either investigate or incorporate this into his or her plans. Educating, identifying, nurturing, and supporting health service planners who have a particular interest in the field may also be a catalyst for change.

The small number of planners interviewed for this study limits the generalisability of the findings; however, participants were all senior planners from a number of Australian states and were all knowledgeable about policy and practice in their state, and well-informed about planning activities within their sector. Each participant, in other words, plays a leading role in health service planning in Australia.

The provision of the background paper to participants before interview listing the health concerns identified in the National Climate Change Adaptation Plan for Human Health (9) may have influenced responses from participants, but if so this would likely be in the direction of participants identifying more, rather than less, health impacts as being of concern to planners.

## Conclusions

Climate change poses considerable challenges and addressing its health consequences requires careful incorporation into health services planning (56). This study highlights the gaps in understanding of health service planners about the health impacts of climate change and suggests ways in which planning for these impacts may start to be addressed.

Nurturing leaders in climate and health planning, incorporating climate data into health service models, and concentrating on planning for services already associated with the highly visible, direct, and readily observable impacts of climate change are all opportunities to begin the process of planning for climate change.

If considered planning is not undertaken, then the Australian health system will only be able to deal with climate change in an expensive ad hoc, crisis management manner. Without meeting the challenge of climate change to the health system head on, Australia will remain unprepared for the health impacts of climate change and this will ultimately have negative consequences for the health of the Australian population.
